# Globular Glial Tauopathy Type I Presenting as Atypical Progressive Aphasia, With Comorbid Limbic-Predominant Age-Related TDP-43 Encephalopathy

**DOI:** 10.3389/fnagi.2019.00336

**Published:** 2019-12-11

**Authors:** Robert Rusina, Zsolt Csefalvay, Gabor G. Kovacs, Jiri Keller, Alena Javurkova, Radoslav Matej

**Affiliations:** ^1^Department of Neurology and Center of Clinical Neuroscience, First Faculty of Medicine, Charles University, and General University Hospital, Prague, Czechia; ^2^Department of Neurology, Third Faculty of Medicine, Charles University and Thomayer Hospital, Prague, Czechia; ^3^Department of Communication Disorders, Comenius University, Bratislava, Slovakia; ^4^Department of Laboratory Medicine and Pathobiology, Faculty of Medicine, University of Toronto, Toronto, ON, Canada; ^5^Tanz Centre for Research in Neurodegenerative Disease, Faculty of Medicine, University of Toronto, Toronto, ON, Canada; ^6^Laboratory Medicine Program & Krembil Brain Institute, University Health Network, Toronto, ON, Canada; ^7^Department of Radiology, Na Homolce Hospital, Prague, Czechia; ^8^Department of Neurology, Third Faculty of Medicine, Charles University, and University Hospital Královské Vinohrady, Prague, Czechia; ^9^Department of Clinical Psychology, Third Faculty of Medicine, Charles University, and University Hospital Královské Vinohrady, Prague, Czechia; ^10^Department of Nursing, Second Faculty of Medicine, Charles University, Prague, Czechia; ^11^Department of Pathology and Molecular Medicine, Third Faculty of Medicine, Charles University and Thomayer Hospital, Prague, Czechia; ^12^Department of Pathology, First Faculty of Medicine, Charles University and General University Hospital Prague, Prague, Czechia

**Keywords:** primary progressive aphasia, tauopathy, globular glial inclusions, TDP-43 proteinopathy, dementia

## Abstract

Globular glial tauopathies (GGTs) have heterogeneous presentations with little available information regarding typical clinical manifestations. We report on a case of atypical primary progressive aphasia (PPA) due to comorbid GGT and limbic transactive response DNA binding protein of 43 kDa (TDP-43) proteinopathy. The initial clinical phenotype was compatible with the nonfluent-agrammatical variant of PPA and early hippocampal amnesia. Progressively, parkinsonism and supranuclear oculomotor impairment occurred, and finally, late mutism with frontal-type dementia, impaired comprehension, and behavioral manifestations developed. The neuropathology was characteristic of GGT type I with vascular changes and comorbid limbic-predominant age-related TDP-43 encephalopathy (LATE). Our findings expand the clinical spectrum of GGTs to include a complex progressive aphasia syndrome. The extraordinary feature, in this case, was the combination of two progressive aphasia subtypes, that is, the early nonfluent-agrammatical variant and the late semantic variant. Our findings also expand the spectrum of neuropathological comorbidities in GGT.

## Background

Globular glial tauopathies (GGTs) are 4R tauopathies, mainly in the form of oligodendroglial and astrocytic inclusions. The most frequent is type I with significant white matter involvement, which has a variable clinical presentation, often mimicking various neurodegenerative diseases, without being type predictive (Kovacs et al., 2008; Ahmed et al., 2013; Graff-Radford et al., 2016).

Transactive response DNA binding protein of 43 kDa (TDP-43) deposits in limbic brain structures are commonly found in the elderly (over 80 years of age). Its clinical impact has been discussed at length, but no widely accepted consensus has been reached. Only recently, a new term was proposed, limbic-predominant age-related TDP-43 encephalopathy (LATE), which encompasses limbic TDP-43 deposits and related cognitive impairment, mainly episodic amnesia mimicking Alzheimer’s disease (Nelson et al., 2019).

GGT has heterogeneous presentations; with little available information regarding typical clinical manifestations. We report on a case of an atypical primary progressive aphasia (PPA) with progressive involvement of both major language streams, that is, the dorsal and ventral pathways, due to GGT and episodic memory loss, due to comorbid LATE neuropathological change (LATE-NC).

We present this complex case from different angles, using a genuinely interdisciplinary approach, documented by repeated assessments, and a focus on the clinical, neuropsychological, language-based, and magnetic resonance imaging (MRI) perspectives.

## Case Presentation

A 69-years-old right-handed man, native Czech speaker, with a history of ischemic stroke in the right central area potentially linked to atrial fibrillation, developed speech difficulties over 1 year. He presented with mild anomia, without language comprehension impairment at the single-word and sentence levels. He had impaired learning ability, executive dysfunction with low verbal and design fluency, and altered motor bimanual and three-stage sequences.

During the second year, he developed lexical retrieval problems, speech apraxia, and agrammatism in spontaneous speech. Comprehension was preserved for single words but was limited for syntactically complex sentences. Intact reading and writing of single content words contrasted with symptoms of phonological alexia and agraphia. Memory impairment worsened, mainly in the form of delayed verbal recall and total verbal learning ability. A neurological examination at this time found a mild resting tremor and right arm bradykinesia.

During the fourth year, dopa-unresponsive right-sided rigidity, hypokinetic dysarthria, and supranuclear oculomotor palsy with slowed voluntary saccades appeared. Oral and written language production and comprehension were impaired at the single-word and sentence levels; few content words remained in his speech.

During the fifth year, he lost comprehension skills and became almost mute, using only nonverbal communication. He also began to express executive and behavioral symptoms, that is, perseveration, emotional blurring, alternating apathy and disinhibition, and episodic compulsive laughter. The patient died later that year.

## Description of Investigations

### Neuropsychology

A detailed neuropsychological assessment was available initially (year 1) and 1 year later (Table 1).

**Table 1:** Neuropsychological assessment.

Neuropsychological tests	RS/WS year 1	Percentile	RS/WS year 2	Percentile
AVLT sum	32	0–10	21	0
AVLT delay	6	10	2	0–10
DS	9	36	7	15
COWAT	10	0	8	0
Design fluency	5	3	2	0
ROCFT immediate	19	41–59	17	60–71
ROCFT delay	19	60–71	22	82–89
ROCFT copy	29	19–28	31	29–40
Picture Compl.	15	95	11	63
BDI-II/GDS	13	minimal	5	low
WAIS-III FIQ	103	58	80	9
WAIS-III VIQ	97	42	63	1
WAIS-III PIQ	110	75	79	8

*AVLT, Auditory Verbal Learning Test (verbal delayed memory and verbal learning); DS, Digit Span Test (immediate memory and attention); ROCFT, Rey–Osterrieth Complex Figure Test (visuospatial functions and visual immediate and delayed memory); COWAT, Controlled Oral Word Association Test (language and executive functions); Design Fluency Test (nonverbal executive function); BDI-II, Beck Depression Inventory, Second Edition (subjective depressive complaints); WAIS-III, Wechsler Adult Intelligence Scale (intellectual abilities: full-scale IQ, verbal IQ, and performance IQ); RS, raw scores; WS, weighted scores*.

At the initial assessment, verbal delayed recall was below average, and overall verbal learning ability was impaired. Executive verbal and design fluency, motor bimanual, and three-stage sequences were impaired, whereas verbal and visual immediate recall, visual delayed recall, visual recognition, and visuospatial skills remained largely preserved. We detected left-sided astereognosia and finger agnosia, but right–left orientation and somatognosis were not affected. The level of depression was minimal, based on his self-assessment. Intellectual abilities were in the average range with a predominance of nonverbal over verbal ones.

During the second year, delayed verbal recall and total verbal learning ability worsened by more than 1 SD. Executive function deterioration was less significant, verbal fluency worsened more (>1 SD) than design fluency (<1 SD), and both verbal recognition and delayed visual recall remained preserved; verbal immediate recall and visual recognition deteriorated less than 1 SD, and visual immediate and delayed recall were in the average. Left-sided astereognosia and finger agnosia remained unchanged. The level of depression was low, based on his self-assessment. There was a general deterioration in his IQ, worsening by more than 1 SD on the overall and verbal parts and decreasing by more than 2 SD on the performance part.

### Language Assessment

The patient initially presented with phonological paraphasias and very mild anomia (problems with lexical retrieval of words with low frequency, late age of acquisition, etc.). There were no repetition errors at the word and sentence levels, nor was there any impairment in single-word or sentence comprehension.

During the second year, a slow increase in lexical retrieval problems in connected speech resulted in more severe anomia (longer pauses in connected speech and longer searches for the correct phonological form of a word) and in agrammatism in spontaneous speech (errors of grammar affecting syntax and the omission of function words). In expression, his spontaneous language production often had simpler syntactic structures, but almost no embedded clauses or incorrect use of grammatical morphemes. Like many languages, Czech has a very rich morphology, and the omission of just one grammatical morpheme in a complex sentence can profoundly change its meaning.

Symptoms of speech apraxia emerged, with marked difficulties in producing polysyllabic words and sequences of syllables on demand that were related to impaired motor programming of speech and reduced articulatory agility.

At this time, he became unable to understand more complex conversations or instructions; however, his recognition of the meaning of single words was still preserved. Normal reading and writing of single content words contrasted with symptoms of phonological alexia and agraphia, which manifested as deficits in the sublexical reading and spelling. While oral reading and reading comprehension of content words were mostly correct, impaired phonological processes compromised his ability to use grapheme/phoneme conversion rules in reading (i.e., oral reading of letters, syllables, and pseudowords) and phoneme/grapheme conversion rules used in writing.

During the fourth year, the patient’s speech production was full of phonetic and articulatory errors and neologisms. He had severe problems even retrieving high-frequency short words and failed to produce sentences with simple syntactic structures (i.e., subject–verb–object); very few content words remained in his speech production. Comprehensive language testing revealed that both oral and written language production and comprehension were severely impaired.

During the fifth year, he completely lost his comprehension skills (i.e., he was unable to correctly solve even very simple high-frequency spoken word–picture or object matching tasks after basic instructions) and became almost mute, using only nonverbal communication, with basic gestures.

### RI Investigations

Three consecutive MRIs (years 1, 2, and 4) were available for analysis. High-resolution 3D T1-weighted images were processed using FreeSurfer^1^. All examinations included fluid-attenuated inversion recovery (FLAIR) sequences; in 2010, the MRI was done using a GE scanner with 5-mm slice thickness and a 1-mm gap; in 2012 and 2014, it was done using a Siemens Avanto scanner with 4-mm slice thickness and a 0.8-mm gap on the middle and a 3D technique (1 × 1 × 1.5 mm) on the last examination. 3D FLAIR was co-registered to an MNI template using the FSL FLIRT tool (Jenkinson and Smith, 2001; Jenkinson et al., 2002), and 2D FLAIR sequences were then co-registered to this volume since a precise co-registration to the final volume was more important than a global template. Then we created a mask covering this area in FslView, calculated both minimum and maximum values for the masked volumes and applied the result to all three examinations as a threshold for co-registered images in order to obtain volumes with clearly pathological signals.

**Figure 1:**
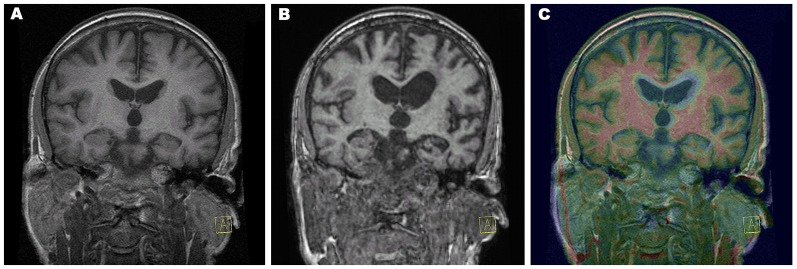
Progression of atrophy rate. Coronal T1-weighted magnetic resonance imaging (MRI) from year 1 **(A)** and from year 4 **(B)** and color fusion of both were performed on a Siemens Leonardo workstation **(C)**. The yellow–red color gradient reflects thresholded T1-weighted image intensity at year 4. Typical white matter signal is red, and regions with longer T1 relaxation time (including gray matter) are yellow. Areas of very long T1 relaxation time (such as the cerebrospinal fluid) are transparent, which clearly depicts the difference between the follow-up image and baseline, which can be seen as a grayscale. Acquisition parameters of 3D T1-weighted sequences: year 1, repetition time (TR) 11.512 ms, echo time (TE) 4.2 ms, inversion time (TI) 500 ms, flip angle (FA) 20, voxel size 0.47 × 0.47 × 2.0, overlap 100%, coronal acquisition; year 4, TR 1,940 ms, TE 3.08 ms, TI 1,100 ms, FA 15, voxel size 0.98 × 0.98 × 1 mm, overlap 0%, sagittal acquisition.

Brain volume and cortical thickness decreased in the left hemisphere (Figure 1) in two different steps. Initially, the atrophy rate progressed more rapidly in the posterior inferior frontal gyrus, Brodmann’s areas 44 and 45, and the Rolandic operculum (between the first and second MRI by 19%; between the first and third MRI by 35%) and to a lesser extent in the paracentral, superior frontal, medial orbitofrontal, and orbital gyri and the insula.

Later, in the progression of the disease, the rapidity of atrophy was greater in the superior temporal gyrus, angular gyrus, and anterior pole of the temporal lobe (between the first and second MRI by 8%; between the first and third MRI by 32%) and to a lesser extent in the temporal pole, middle temporal gyrus, and lateral occipitotemporal sulcus).

**Figure 2:**
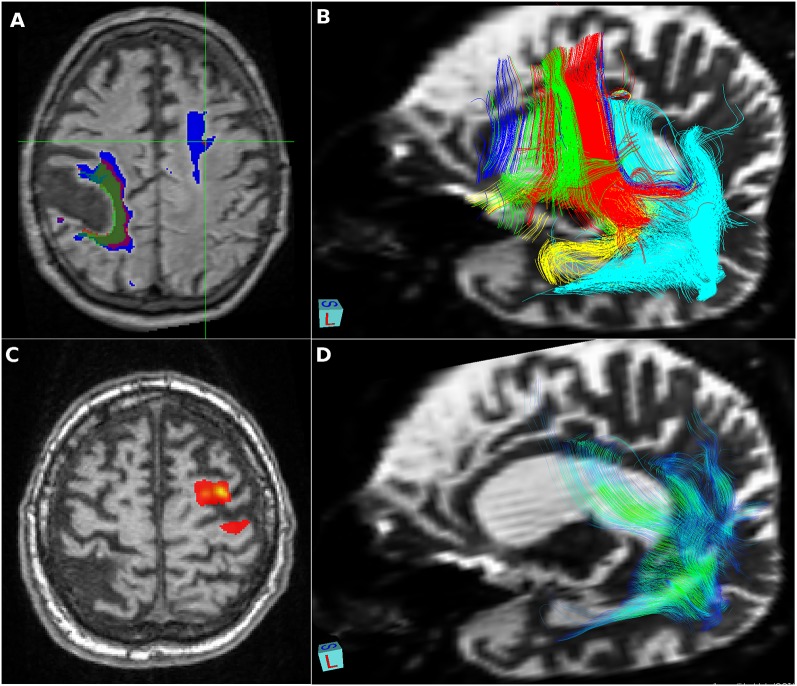
Vascular lesions and tractography—visualization provided using FSLeyes, part of FSL, and deterministic tractographies processed in MedINRIA 1.9.2. **(A)** Relative increase in size of a right-sided residual poststroke lesion (2010 green, 2012 red, and 2014 blue) and significant growth of the fluid-attenuated inversion recovery (FLAIR) left frontal white matter hyperintensity detected in 2012; **(B)** deterministic tractography of the dorsal stream: fibers originating from Brodmann’s areas BA44 in red, BA45 in green, inferior frontal gyrus in blue, insula in yellow, superior temporal gyrus in turquoise, and precentral gyrus in violet; **(C)** probabilistic tractography shows only local connectivity to the precentral gyrus; **(D)** deterministic tractography of the ventral stream: fibers originating from the angular gyrus in color reflecting the value of local fractional anisotropy.

Further, we analyzed associated vascular lesions in the brain (Figure 2). A large residual poststroke lesion in the right central lesion increased in volume over time, related to adjacent brain atrophy. We also detected in year two a FLAIR hyperintensity in the white matter of the left frontal lobe that increased in size significantly in year 4.

Hippocampal atrophy also increased over time, assessed using the MTA (medial temporal atrophy scale; Scheltens et al., 1992): in year 1, MTA 0–1; in year 2, MTA left 2, right 1; in year 4, MTA left 4, right 2.

### Neuropathology

**Figure 3:**
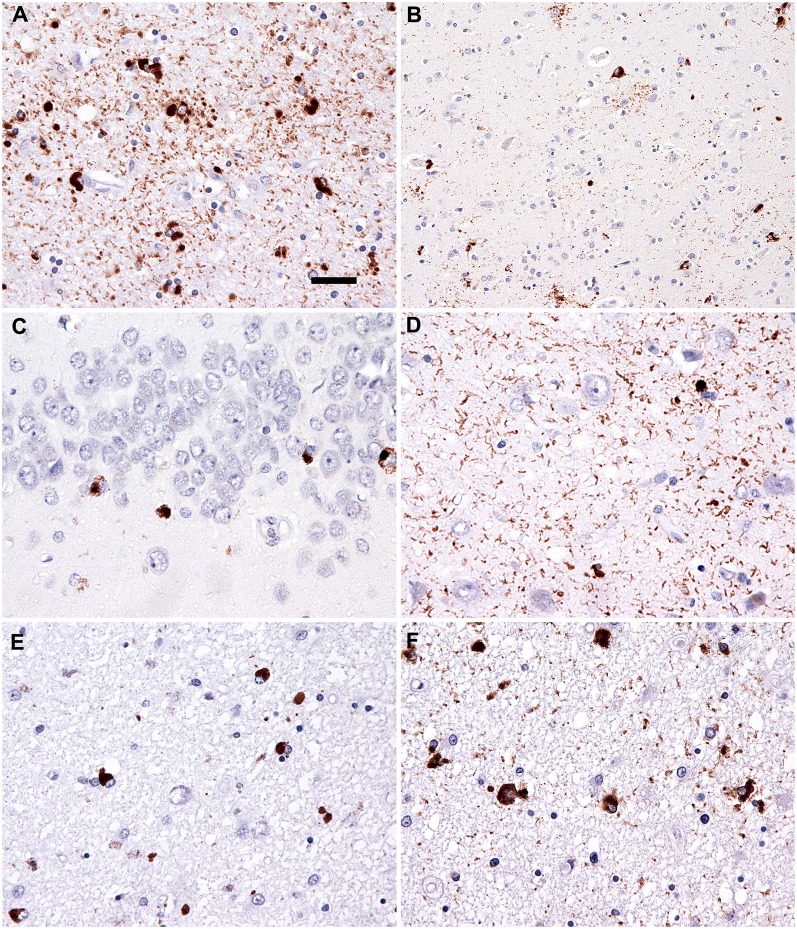
Neuropathology. Immunostaining for phospho-Tau (AT8) reveals characteristic globular oligodendroglial inclusions in the white matter (**A**; hippocampus) and astroglial and less neuronal tau pathology in the gray matter (**B**; temporal cortex). Immunostaining for phospho-transactive response DNA binding protein of 43 kDa (TDP-43) reveals occasional neuronal cytoplasmic inclusions in the granule cells of the dentate gyrus **(C)** together with abundant fine threads in the hippocampus **(D)**. Interestingly, few oligodendroglial inclusions with globular morphology were noted in the phospho-TDP-43 (**E**; peri-amygdala white matter) immunostaining reminiscent of the tau pathology (**F**; corresponding area to that shown in **E**). Bar in **(A)** represents 25 μm for **(D–F)** and 50 μm for **(B,C)**.

On autopsy, disperse vascular changes of different degrees were combined with neuronal loss, gliosis and spongy vacuolation of the superficial layers were seen in the frontal and temporal cortices. Immunohistochemistry for hyperphosphorylated tau protein (Figures 3A,B) detected numerous argyrophilic oligodendroglial globular inclusions, white matter granular deposits, and globular non-argyrophilic astroglial inclusions; neurons showed diffuse cytoplasmic staining and inclusions. Tau pathology was mostly 4R, with rare, unusual 3R oligodendroglial immunoreactivity, as reported earlier (Ferrer et al., 2014). The observed neuropathological patterns fulfilled the diagnostic criteria of GGT type I (Ahmed et al., 2013).

Moreover, the hippocampuss and amygdala showed phospho-TDP-43 neuronal cytoplasmic inclusions, fine threads, and occasional small globular oligodendroglial immunoreactivity (Figures 3C–F) associated with moderate segmental gliosis in the CA1 subregion. Due to the restricted neuroanatomical distribution and the lack of involvement of the frontal and/or temporal lobes, the TDP-43 pathology met the criteria for LATE-NC, a recently proposed term to describe a limbic predominant neurodegenerative entity with TDP-43 pathology (Nelson et al., 2019). Other neuropathological hallmarks of concomitant neurodegenerative disorder, namely Alzheimer’s disease, were excluded during complex brain tissue investigations. Molecular genetic analysis of crucial genes involved in the pathophysiology of frontotemporal lobar degenerations (*MAPT*, *PS1*, *PS2*, *PGRN*, and *TARDBP*) did not reveal any pathological gene aberrancies.

## Discussion

We present a case with early nonfluent PPA and hippocampal amnesia, progressive parkinsonism and supranuclear oculomotor impairment, and finally, late development of mutism with frontal-type dementia, impaired language comprehension, and behavioral manifestations. The neuropathological background shows a rare association between GGT type I and hippocampal sclerosis, with features of LATE-NC and vascular encephalopathy.

While most PPA patients fit into one of three main clinical syndromes [the nonfluent-agrammatical (nfvPPA), semantic (svPPA), and logopenic variants], some patients meet the criteria for more than one variant or present with mixed cognitive–anatomical deficits during the disease course (De Leon et al., 2019). Since specific symptoms are related to damage to certain brain networks, variable speech and language impairments caused by degeneration of multiple networks can occur in the presence of a mixed pathology. The clinical spectrum of nfvPPA is the most diverse of the canonical PPA syndromes, with a number of variant subsyndromes (Marshall et al., 2018).

The language-relevant brain regions, that is, Broca’s area in the inferior frontal cortex and Wernicke’s area in the superior temporal cortex, are connected *via* long fiber bundles, which are located dorsally and ventrally to the Sylvian fissure. These dorsal and ventral pathways consist of a number of partially parallel fiber tracts, which can be differentiated by their termination regions and by the particular language functions of these termination regions (Friederici, 2015).

Our patient initially met the clinical criteria of nfvPPA (Gorno-Tempini et al., 2011), including agrammatism and mild speech apraxia with impaired syntax processing and spared single-word comprehension and semantics. Severe impairment of phonological processes can be attributed to a breakdown of the dorsal stream in the language-dominant hemisphere: lesions to the left perisylvian cortical regions, including the inferior frontal gyrus, Rolandic operculum, precentral gyrus, insula, supramarginal gyrus, and superior temporal gyrus (Henry et al., 2016), and/or damage to both major fiber tracts connecting the posterior temporal cortex with the frontal cortex terminating in the premotor cortex (supporting sensory-to-motor mapping) and in posterior Broca’s area and the pars opercularis (supporting the processing of complex syntactic structures; Friederici, 2015). In our patient, the early and rapid progression of atrophy corresponded to structures forming the dorsal stream (Figure 2). Our patient also developed a subcortical vascular FLAIR hyperintensity in the dominant frontal lobe, which increased in size over time and initially predominantly affected fibers to the precentral gyrus (Figure 2). This finding, in line with reported white matter lesion in the GGT type I, struck the dorsal language stream and could have had a severe impact on syntax deficits and frontal inhibition late in the disease course. Moreover, in nfvPPA cases (with similar findings to ours), tauopathy-related parkinsonism and supranuclear gaze palsy are often reported (Rohrer et al., 2010; Montembeault et al., 2018).

With gradual disease progression, severe oral and written language comprehension deficits developed at the single-word level, which could be attributed to damage to the language-related fiber tracts of the ventral stream that is suggested to support semantic processes (Friederici, 2015). These severe language problems combined with behavioral manifestations (disinhibition, compulsive laughter, and blurred emotion) and slowly progressing focal MRI atrophy of the dominant angular gyrus, superior temporal gyrus, and temporal pole, finally progressing to severe frontal dementia with mutism, strongly evoked the semantic variant of PPA (svPPA; Gorno-Tempini et al., 2011; Henry et al., 2016). This is in line with MRI findings in our patient, in particular, the late onset of rapidly increasing atrophy in structures forming the ventral stream including the anterior and inferolateral temporal lobes and angular gyrus (Figure 1).

The patient’s cognitive assessment also showed obvious early memory loss; the impaired verbal learning ability was in line with marked hippocampal atrophy (Figure 1). These findings could be attributable to comorbid LATE, which is associated with amnestic dementia mimicking Alzheimer’s disease and atrophy in the medial temporal lobes and frontal cortex (Nelson et al., 2019), even though LATE is more typical in elderly patients. There were no neuropathological hallmarks of concomitant Alzheimer’s disease present. The observed parietal manifestations (astereognosis and left-handed finger agnosia) can be attributed to a large residual ischemic lesion in the right central area, unrelated to either the GGT or LATE inclusions.

Neuropathology was characteristic of GGT type I, with LATE-NC combined with vascular changes of variable severity. The discussed dorsal and ventral stream interruption corresponds, from a neuropathology point of view, to destruction of cortical lesions by neuronal cell loss and concomitant damage to long fiber bundles due to severe white matter involvement (pathognomonic for GGT type I; Ahmed et al., 2013). Hippocampal tissue analysis showed circumscribed involvement of the subiculum as seen in pure GGT type I cases (subiculum), with the additional gliotic focus in the CA1 with TDP-43 deposits being compatible with LATE-NC (Nelson et al., 2019).

Both nfvPPA and svPPA have been previously, but only separately, reported in the GGT pathology (Ahmed et al., 2013; Graff-Radford et al., 2016; Kim et al., 2017). TDP-43 pathology was described in a single case of GGT type III (Takeuchi et al., 2016), but not for GGT type I. According to an observation by Takeuchi et al., the TDP-43 pathology is different from typical LATE-NC and shows co-localization of tau and TDP-43 in some inclusions (Takeuchi et al., 2016).

In summary, our findings expand (1) the clinical spectrum of GGTs to include a complex progressive aphasia syndrome (a very unusual feature, in this case, is the combination of two PPA subtypes, early nfvPPA and late svPPA) and (2) the spectrum of neuropathological comorbidities in GGT with concomitant LATE-NC.

## Data Availability Statement

The datasets generated for this study are available on request to the corresponding author.

## Ethics Statement

This study was approved by the Multicenter Ethics Committee, the Institute of Clinical and Experimental Medicine and Thomayer Hospital, Prague, Czech Republic. Publication consent was obtained from the relatives of the patient.

## Author Contributions

RR was involved in the study concept and design, interpretation of results, study supervision, and drafting/revising the manuscript. RM was involved in the study concept, manuscript drafting, and revising. GK, ZC, JK and AJ were involved in data acquisition and analysis and interpretations of results. ZC, GK and JK were involved in the critical revision of the manuscript.

## Conflict of Interest

The authors declare that the research was conducted in the absence of any commercial or financial relationships that could be construed as a potential conflict of interest.

## Footnotes

^1^http://surfer.nmr.mgh.harvard.edu

## Abbreviations

GGT: globular glial tauopathies
LATE: limbic-predominant age-related TDP-43 encephalopathy
LATE-NC: LATE neuropathological change
MT: medial temporal atrophy scale
nfvPPA: progressive aphasia (nonfluent-agrammatical variant)
svPPA: progressive aphasia (semantic variant)
TDP-43: transactive response DNA binding protein of 43 kDa.
